# Systematic evaluation of the adaptability of the non-radioactive SUnSET assay to measure cardiac protein synthesis

**DOI:** 10.1038/s41598-018-22903-8

**Published:** 2018-03-15

**Authors:** Venkatraman Ravi, Aditi Jain, Faiz Ahamed, Nowrin Fathma, Perumal Arumugam Desingu, Nagalingam R. Sundaresan

**Affiliations:** 1 Cardiovascular and Muscle Research Laboratory, Department of Microbiology and Cell Biology, Indian Institute of Science, Bengaluru, India; 2 Centre for BioSystems Science and Engineering, Indian Institute of Science, Bengaluru, India

## Abstract

Heart is a dynamic organ that undergoes remodeling in response to both physiological and pathological stimuli. One of the fundamental cellular processes that facilitates changes in the size and shape of this muscular organ is the protein synthesis. Traditionally changes in cardiac protein synthesis levels were measured by radiolabeled tracers. However, these methods are often cumbersome and suffer from radioactive risk. Recently a nonradioactive method for detecting protein synthesis under *in vitro* conditions called the Surface Sensing of Translation (SUnSET) was described in cell lines of mouse dendrites and T cells. In this work, we provide multiple lines of evidence that the SUnSET assay can be applied to reliably detect changes in protein synthesis both in isolated neonatal primary cardiomyocytes and heart. We successfully tracked the changes in protein synthesis by western blotting as well as immunohistochemical variants of the SUnSET assay. Applying the SUnSET assay, we measured the cardiac protein synthesis during the different ages of mice. Further, we successfully tracked the increase in cardiac protein synthesis during different stages of a well-established model for pathological hypertrophy. Overall, we propose SUnSET assay as a simple, reliable and robust method to measure protein synthesis in the cardiac milieu.

## Introduction

Mammalian heart is a dynamic organ, which undergoes frequent changes in the overall size and shape in response to varying workloads^1^. The heart is predominantly composed of cardiomyocytes, which are terminally differentiated cells with limited dividing potential^2^. Any increase in the size of this muscular organ is therefore brought about by enlargement of the individual cardiomyocytes, a process termed as hypertrophy^3^. One of the fundamental cellular mechanisms that facilitate the hypertrophic enlargement of the heart is the protein synthesis, which makes new proteins to support the increase in the size of individual cardiomyocytes^4^. Even though the protein synthesis rates in the adult heart are the lowest amongst various tissues, it demonstrates remarkable capability to be stimulated in response to various hypertrophic cues^5^.

Normal physiological enlargement of the heart in response to exercise does not usually contribute to the risk of heart failure. However, under pathological conditions, initially adaptive hypertrophic response turns maladaptive and significantly increases the risk of cardiovascular dysfunction and mortality^6^. Pathological cardiac hypertrophy is thus a major factor that predisposes individuals to heart failure. Currently heart failure is one of the leading causes of death worldwide affecting an estimated 38 million patients across the globe^7^. The study of alterations in protein synthesis in the context of hypertrophy thus assumes special significance. It helps delineate the mechanisms behind the regulation of this complex and fundamental cellular process and devise interventions to overcome the complications in heart failure.

Traditionally protein synthesis in heart has been studied in experimental models by tracking the incorporation of radiolabeled amino acid tracers into the newly synthesized proteins. Despite their wide use and versatility, these methods are often expensive, time consuming and carry potential risk from the use of radioactive substances^8^. Recently, a nonradioactive method for assessing protein synthesis in cell cultures known as the surface sensing of translation (SUnSET) was developed and validated against the existing methods^9^. This method relies on the principle that puromycin, an amino-nucleoside antibiotic produced by *Streptomyces alboniger*, is incorporated into elongating peptide chains at low concentration, owing to its structure analogy to tyrosyl t-RNA^10^. At high concentration, puromycin effectively shuts down translation. However, at very low concentrations and brief time periods, it neither interferes with the overall translation rates nor induces a stress response. The puromycin conjugated peptides can then be detected immunologically using an anti-puromycin antibody, which represents the overall rate of protein synthesis^9^. This technique has been adapted to muscle tissue fibers, hippocampal sections and *Arabidopsis* roots for measuring protein synthesis^11–13^. However, this method has not been studied systematically to measure the alterations in cardiac protein synthesis. Owing to our ongoing interest in studying regulatory mechanisms of cardiac hypertrophy and protein synthesis, we evaluated the suitability and the appropriate use of the SUnSET assay for isolated neonatal cardiomyocytes and murine hearts.

## Results

### Evaluation of SUnSET assay in primary cardiomyocyte cell culture

**Figure 1:**
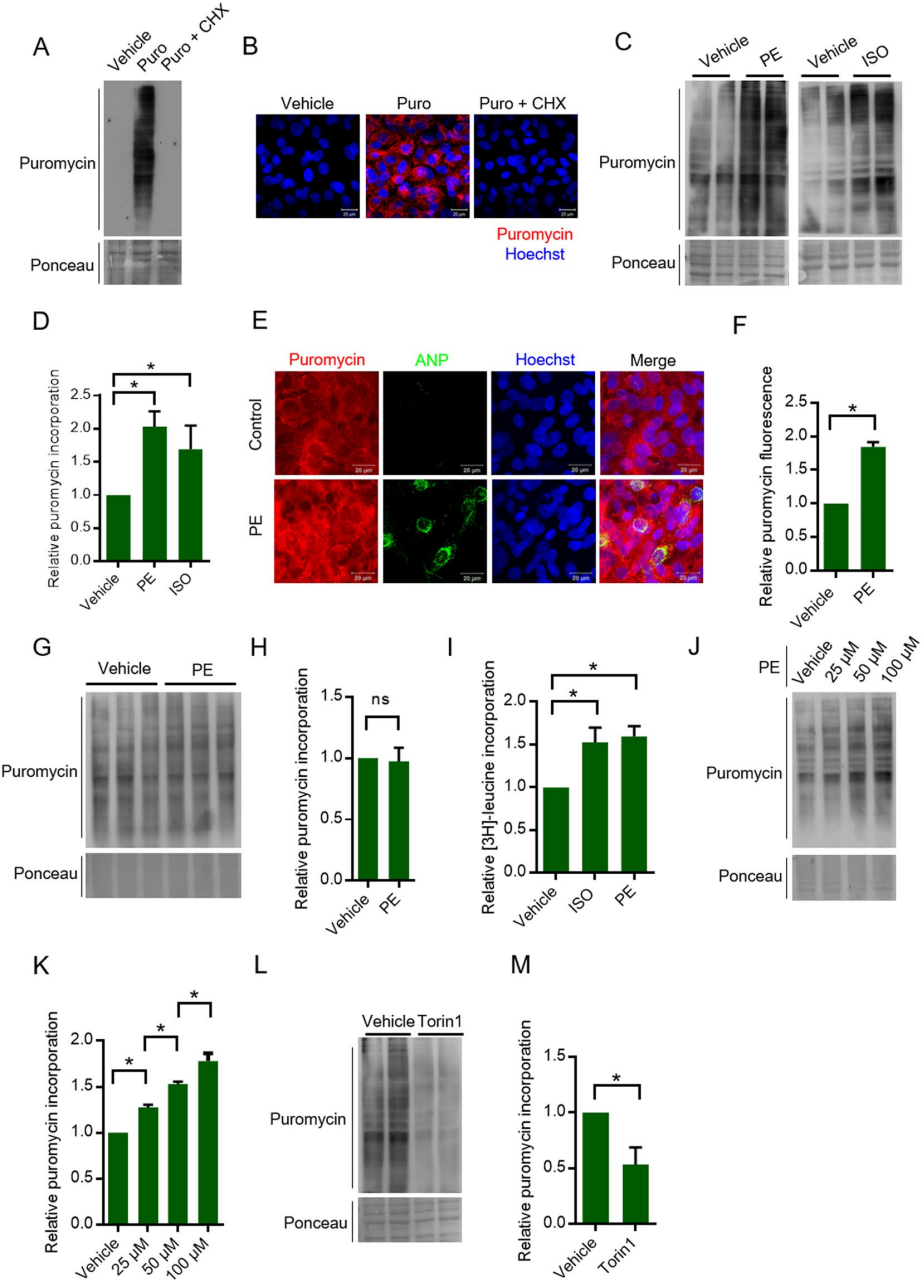
(**A**) Representative images of WB-SUnSET analysis of neonatal rat cardiomyocytes treated with vehicle or puromycin (Puro) or puromycin and cycloheximide (CHX). Ponceau staining was used to confirm equal loading. n = 3 independent experiments. (**B**) Representative images IFC-SUnSET analysis of neonatal rat cardiomyocytes treated with vehicle or puromycin (Puro) or puromycin and cycloheximide (Puro + CHX). Puromycin staining is shown in red and the nuclei stained with Hoechst 33342 are shown in blue. Scale bar = 20 µm. n = 3 independent experiments. (**C**) Representative images of WB-SUnSET analysis of neonatal rat cardiomyocytes treated with adrenergic receptor agonists ISO and PE. The changes in protein synthesis were compared against vehicle treated cells and equal loading was confirmed with Ponceau staining. (**D**) Quantitative representation of puromycin incorporation observed in Fig. 1C. The results are expressed as the fold change relative to vehicle treated cells. n = 4 to 5 independent experiments. Data are presented as mean ± s.d, *p < 0.05. (**E**) Representative images of IFC-SUnSET analysis of vehicle or PE treated cardiomyocytes. Puromycin staining is shown in red, ANP is shown in green and the nuclei are stained with Hoechst 33342 and shown in blue. Scale bar = 20 µm. (**F**) Quantitative representation of relative puromycin fluorescence intensity as observed in Fig. 1E. The results are expressed as fold change relative to vehicle treated cells. For quantification, 50–100 cells were used for each group. Data are presented as mean ± s.d, *p < 0.05. (**G**) Representative images of WB-SUnSET analysis of neonatal rat myotubes treated with PE. The changes in protein synthesis were compared against vehicle treated cells and equal loading was confirmed with Ponceau staining. (**H**) Quantitative representation of puromycin incorporation observed in Fig. 1G. The results are expressed as the fold change relative to vehicle treated cells. n = 6 independent experiments. Data are presented as mean ± s.d, ns – not significant. (**I**) Quantitative representation of changes in protein synthesis upon ISO and PE treatment measured by [^3^H]-leucine incorporation assay. The results are expressed as the fold change relative to vehicle treated cells. n = 4 to 5 independent experiments. Data are presented as mean ± s.d, *p < 0.05. (**J**) Representative images of WB-SUnSET analysis of neonatal rat cardiomyocytes treated with different doses of PE between 0–100 µM. The changes in protein synthesis were compared against vehicle treated cells and equal loading was confirmed with Ponceau staining. (**K**) Quantitative representation of puromycin incorporation observed in Fig. 1J. The results are expressed as the fold change relative to vehicle treated cells. n = 3 independent experiments. Data are presented as mean ± s.d, *p < 0.05. (***L***) Representative images of WB-SUnSET analysis of neonatal rat cardiomyocytes treated with vehicle or Torin1. Equal loading was confirmed with Ponceau staining. (**M**) Quantitative representation of puromycin incorporation as observed in Fig. 1L. The results are expressed as fold change relative to vehicle treated cells. n = 4 independent experiments. Data are presented as mean ± s.d, *p < 0.05.

To test the application of the SUnSET assay for measuring protein synthesis under *in vitro* conditions, we used primary cultures of neonatal rat cardiomyocytes. Immunoblotting analysis of the puromycin labeled cardiomyocyte lysates displayed a trail of puromycin labelled peptides, when probed with a monoclonal antibody against puromycin (Fig. 1A). However, vehicle treated cells did not display puromycin signal, when probed with the same antibody (Fig. 1A). Furthermore, treatment of the cardiomyocytes with the translation inhibitor cycloheximide along with the pulse of puromycin prevented puromycin incorporation at global level (Fig. 1A). These evidences suggest that the puromycin antibody specifically detects the puromycin incorporated in the newly synthesized nascent polypeptide chains only. Further it rules out the possibility of puromycin cross reacting with any existing proteins inside the cell. We observed similar results when we tracked puromycin incorporation by immunofluorescence microscopy as well, indicating the adaptability of the basic principle across multiple detection methods (Fig. 1B).

Protein synthesis in cardiomyocytes is known to be up-regulated in response to various hypertrophic agents such as Isoproterenol (ISO) and Phenylephrine (PE), which are β and α adrenergic receptor agonists respectively^5,14^. We treated the cardiomyocytes with these agents and assayed the changes in protein synthesis by western blotting SUnSET (WB-SUnSET) and immunofluorescence SUnSET (IFC-SUnSET). We observed a significant increase in puromycin incorporation following ISO or PE treatment using the WB-SUnSET assay (Fig. 1C,D). We observed a similar increase in protein synthesis when the cardiomyocytes were treated with PE and evaluated by IFC-SUnSET assay (Fig. 1E,F). In addition, the PE treated cardiomyocytes displayed increased expression of ANP which is a characteristic marker of fetal gene reprogramming, thus confirming the induction of hypertrophy (Fig. 1E)^15^. However, we did not observe any significant changes in the overall protein synthesis levels, when we treated primary cultures of differentiated myotubes with PE (Fig. 1G,H). Next, we tested the efficiency of SUnSET assay in comparison to the classical [^3^H]-leucine incorporation assay. Notably, the changes in protein synthesis rates observed by the standard [^3^H]-leucine incorporation assay were comparable to those obtained by SUnSET assay (Fig. 1I). This indicates that the SUnSET assay is a sensitive and reliable alternative to classical [^3^H]-leucine incorporation assay.

Further to demonstrate the ability of the assay to detect dose dependent changes in protein synthesis, we treated primary cardiomyocytes with different concentrations of PE between 0–100 µM. We indeed observed a steady dose dependent increase in protein synthesis within the range tested, indicating that the assay is sensitive enough to detect even small incremental changes (Fig. 1J,K).

Previous studies suggest that one of the key regulators and an activator of protein synthesis inside the cell is the mTOR signaling pathway^16^. Treatment of cells with Torin1, an ATP competitive inhibitor of the mTOR protein is known to attenuate protein synthesis^17^. We tested whether inhibition of protein synthesis on treatment with Torin1 could be explained by SUnSET assay. WB-SUnSET analysis of neonatal cardiomyocytes treated with Torin1 displayed decreased puromycin incorporation as compared to the controls, indicating the inhibition of protein synthesis in these cells (Fig. 1L,M). These results demonstrate the validity of this method in detecting changes in protein synthesis under diverse conditions in primary cardiac cell culture model.

### Measurement of *in vivo* cardiac protein synthesis using SUnSET assay

**Figure 2:**
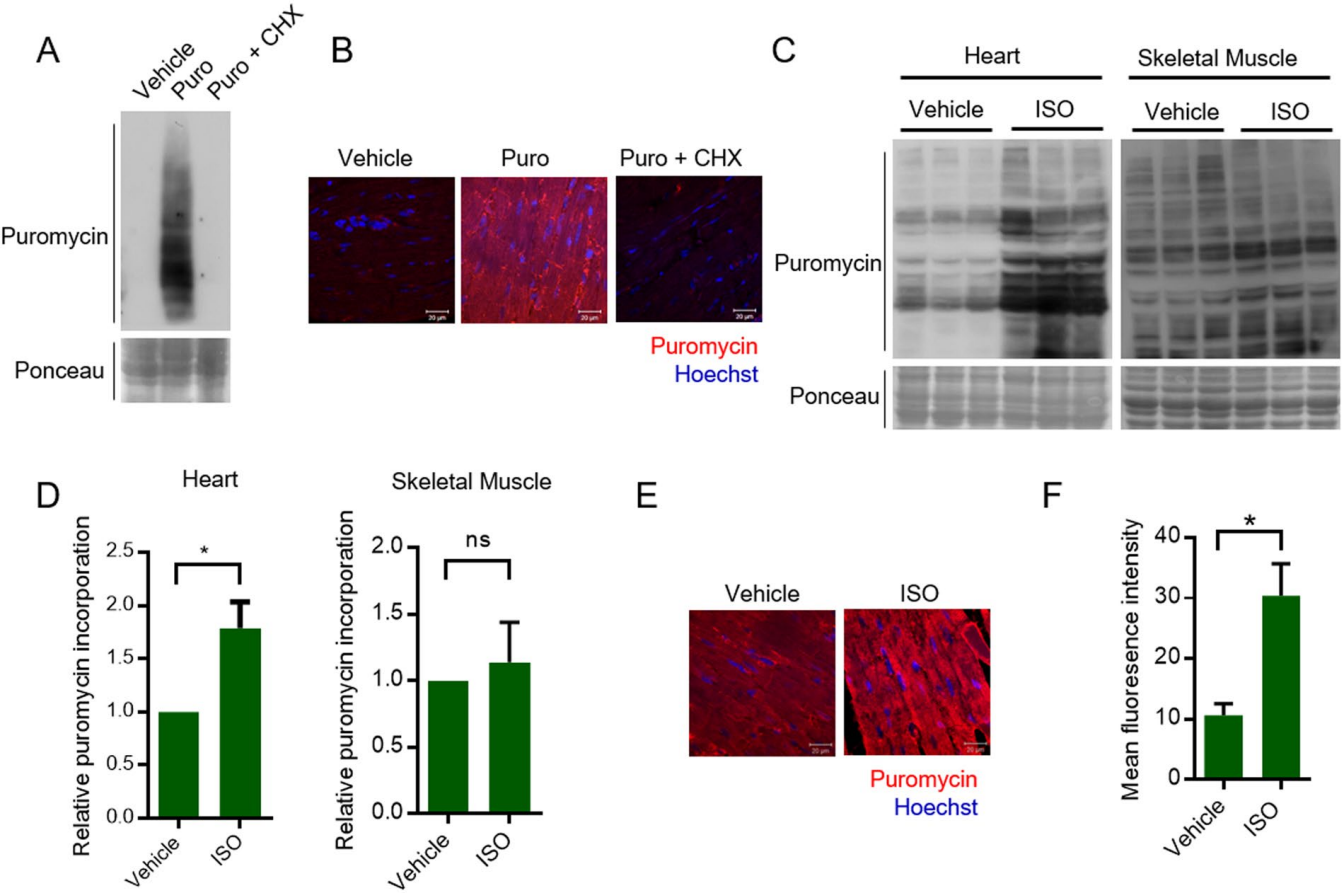
(**A**) Representative images of WB-SUnSET analysis of heart tissues lysates isolated from mice injected with vehicle or puromycin (Puro) or puromycin and cycloheximide (Puro + CHX). Ponceau staining was used to confirm equal loading. (**B**) Representative images of formalin fixed cardiac tissue sections from mice injected with vehicle or puromycin or puromycin and cycloheximide. The sections were immunostained with anti-puromycin antibody (Red) and visualized by confocal microscopy. The nuclei were stained with Hoechst 33342 (Blue). Scale bar = 20 µm. (**C**) Representative images of WB-SUnSET analysis of heart and skeletal muscle lysates from vehicle or ISO treated mice. Equal loading was confirmed with Ponceau staining. (**D**) Quantitative representation of puromycin incorporation as observed by WB-SUnSET in heart and skeletal muscle tissues upon ISO treatment as represented in Fig. 2C. The results are expressed as the fold change relative to vehicle injected mice. n = 6 mice per group. Data are presented as mean ± s.d, *p < 0.05, ns – not significant. (**E**) Representative images of IHC-SUnSET analysis of control or ISO treated mice cardiac tissue sections. Puromycin staining is shown in red and the nuclei stained with Hoechst 33342 is shown in blue. Scale bar = 20 µm. (**F**) Quantitative representation of changes in puromycin fluorescence intensity observed in Fig. 2E. The results are expressed as mean fluorescence intensity values calculated from multiple fields acquired from tissue section across three individual groups. Data are presented as mean ± s.d, *p < 0.05.

We next tested the suitability of this assay to measure *in vivo* changes in protein synthesis using mice model. Western blotting analysis suggested puromycin incorporation in heart tissue lysate of mice after intraperitoneal injection of puromycin (Fig. 2A). Similar to *in vitro* conditions, we did not observe any signal against puromycin antibody in vehicle injected mice or in mice injected with cycloheximide, prior to puromycin administration (Fig. 2A,B). Next, to study the relative changes in protein synthesis, the mice were injected with either vehicle or ISO. The puromycin incorporation rates were markedly high in heart tissues of ISO treated mice when compared to control mice, when analyzed by both western blotting as well as immunohistochemistry (IHC-SUnSET) variants of the assay (Fig. 2C,D,E and F). Further, we analyzed the effects of ISO on skeletal muscle tissue under *in vivo* conditions. Although, there appears to be increased puromycin incorporation in some proteins of skeletal muscle tissue of ISO administered mice, we do not see significant changes in the overall protein synthesis levels, consistent with the previously reported effects of ISO on skeletal muscle (Fig. 2C,D)^18^. This further highlights the versatility of the method in detecting organ specific changes in protein synthesis.

### SUnSET assay accurately measures the increase in rate of protein synthesis during pathological hypertrophy

**Figure 3:**
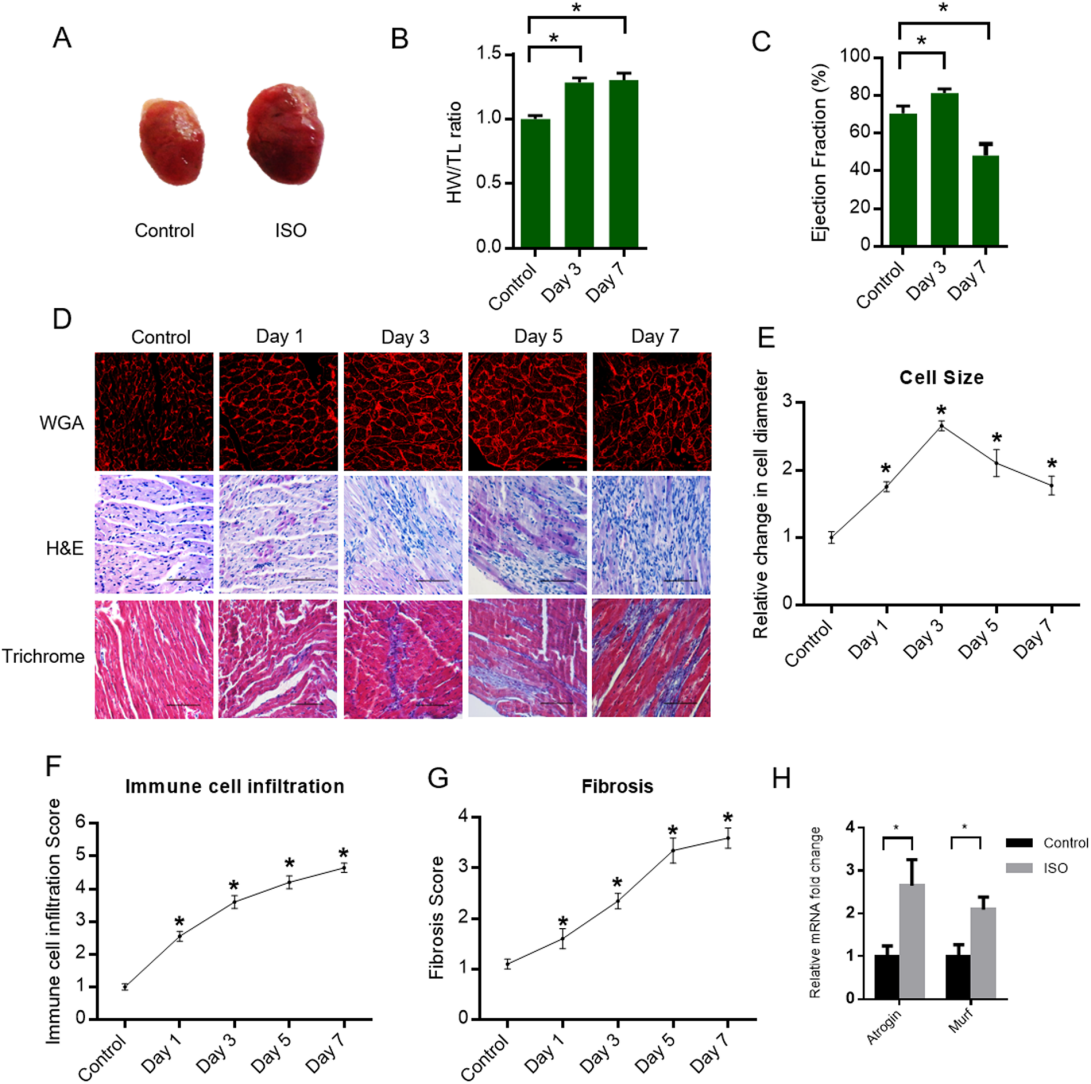
(**A**) Representative images of the hearts collected from vehicle injected mice or mice administered with ISO for 7 days. (**B**) Heart weight to tibia length ratio, represented as the mean ± s.d for each group. n = 4 mice per group. *p < 0.05. (**C**) Cardiac function assessed in control and ISO treated mice by measuring ejection fraction using echocardiography. The results are expressed as the mean ± s.d for each group. n = 4 mice per group. *p < 0.05. (**D**) Representative images of cardiac tissue sections stained with WGA or H&E or Masson’s Trichrome staining from mice treated with ISO for different time periods. Scale bar = 20 µm for WGA sections. Scale bar = 1 mm for H&E and Trichrome staining. (**E**) Quantitative representation of changes in cell size as observed by WGA staining in Fig. 3D. The results are expressed as the fold change relative to vehicle injected mice (Control). n = 4 mice per group. Data are presented as mean ± s.d, *p < 0.05. Significance was calculated with reference to control mice. (**F**) Immune cell infiltration was scored on a scale of 1 to 5 from the H&E stained cardiac tissue sections as presented in Fig. 3D. n = 4 mice per group. Data are presented as mean ± s.d, *p < 0.05. Significance was calculated with reference to control mice. (**G**) Fibrosis was scored on a scale of 1 to 5 from the Trichrome stained cardiac tissue sections as presented in Fig. 3D. n = 4 mice per group. Data are presented as mean ± s.d, *p < 0.05. Significance was calculated with reference to control mice. (**H**) RT-PCR analysis of cardiomyocyte atrophy markers Atrogin and Murf in vehicle (Control) or ISO injected (5 days) mice. The results are expressed as the fold change relative to control group. n = 3 mice per group. Data are presented as mean ± s.d, *p < 0.05.

Chronic administration of ISO is known to induce cardiac hypertrophy and ultimately lead to cardiac failure^19^. To systematically evaluate the changes in protein synthesis accompanying the development of cardiac hypertrophy, we treated mice with ISO for seven days at specific intervals. We observed that the heart size was appreciably larger after seven days of ISO treatment (Fig. 3A). Also, the heart weight to tibia length ratio was significantly higher from the third day of ISO treatment compared to the control group (Fig. 3B). Next, we performed echocardiography to test the effect of ISO on cardiac function. We observed increased ejection fraction on third day of treatment, indicating a compensatory effect on the heart. However, we observed significantly reduced ejection fraction on seventh day of ISO treatment, indicating contractile dysfunction (Fig. 3C). Further, we compared morphological changes in heart of ISO treated mice. To evaluate the changes in cardiomyocyte-size, we performed WGA staining in heart tissue sections injected with ISO. WGA staining suggested that ISO treatment significantly increases the cross-sectional area of cardiomyocytes in the initial stages, which is a hallmark of hypertrophy (Fig. 3D,E). Though the cell size decreased in the later stages of ISO treatment, the heart weight was largely preserved (Fig. 3B). This could be due to extensive cardiac remodeling involving a fibrotic response and immune cell infiltration as observed in the histological examinations (Fig. 3D). It is well established that prolonged ISO administration is cardiotoxic and induces myocardial necrosis^20–22^ and increased cardiac fibrosis^23,24^. To verify this, we performed H&E staining, Masson’s trichrome staining and TUNEL assay in the cardiac tissue sections of ISO administered mice. These histological examinations revealed profuse immune cell infiltration, severe interstitial as well as replacement fibrosis and cardiomyocyte apoptosis with the severity increasing with the duration of ISO treatment (Fig. 3D,F and G and Supplementary Fig. 1A,B). The degenerative changes observed by histological analysis were scored on a scale of 1 to 5 in a blinded fashion (Fig. 3F,G)^25,26^. As a further validation, we quantified the expression of Atrogin-1 and MuRF1, ubiquitin ligases, which are responsible for the cardiac muscle atrophy^27,28^. Our results suggest that the expression levels of ubiquitin ligases, Atrogin-1 and MuRF-1, are increased in ISO treated heart samples (Fig. 3H). This data confirms the atrophic changes in heart of ISO treated mice. These evidences clearly establish the development of cardiac hypertrophy, fibrosis and ultimately cardiac failure in mice upon ISO administration.

**Figure 4:**
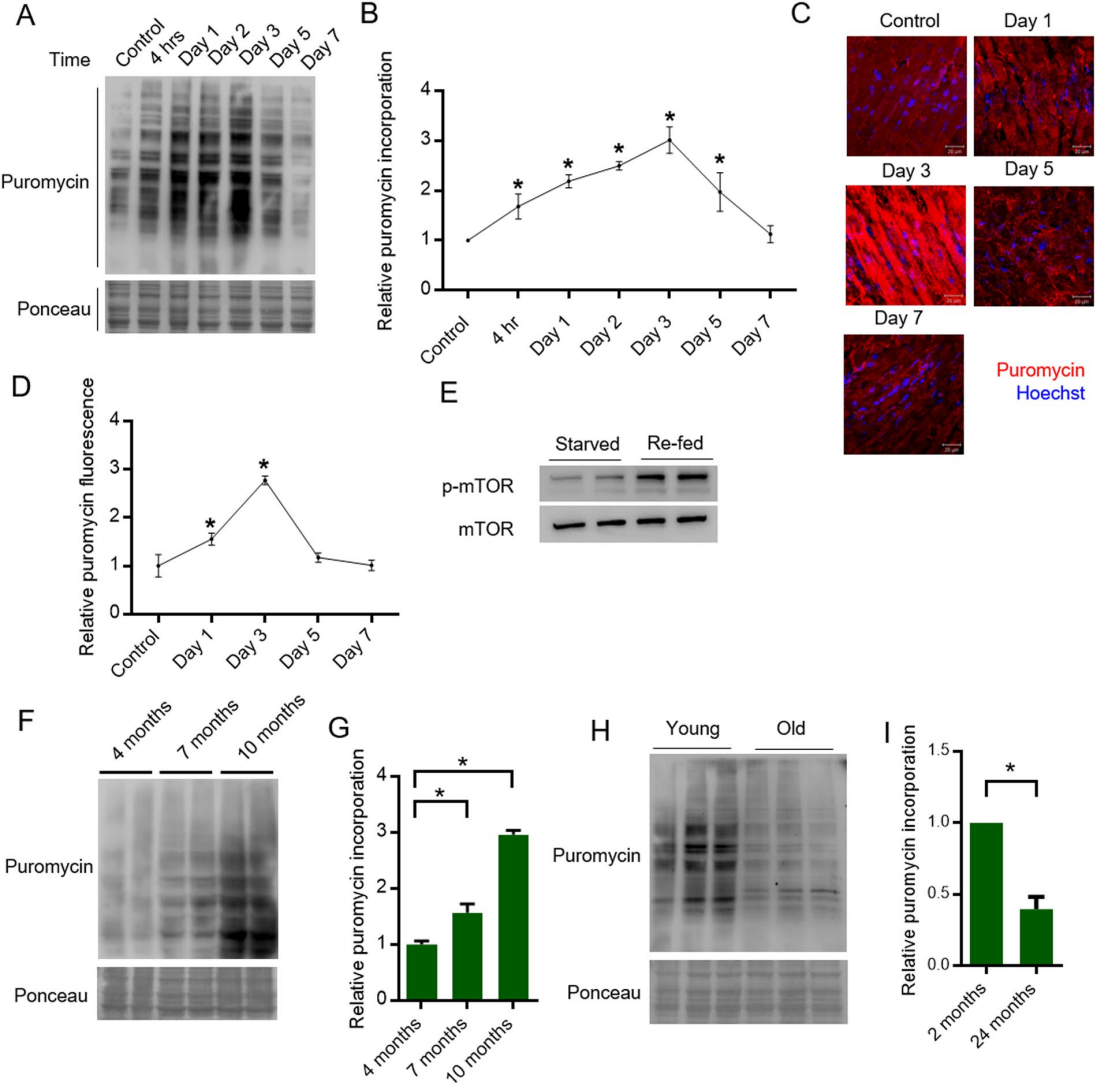
(**A**) Representative images of WB-SUnSET analysis of heart tissues lysates from mice injected with ISO for different time periods between 0–7 days. Ponceau staining was used to confirm equal loading. (**B**) Quantitative representation of puromycin incorporation observed in Fig. 4A. The results are expressed as the fold change relative to control mice. n = 3 to 4 mice per group. Data are presented as mean ± s.d, *p < 0.05. Significance was calculated with reference to control mice. (C) Representative images of IHC-SUnSET analysis of heart tissues sections from mice injected with ISO for different time periods between 0–7 days. The sections were immunostained with anti-puromycin antibody (Red) and the nuclei were stained with Hoechst 33342 (Blue) and visualized by confocal microscopy. Scale bar = 20 µm. (**D**) Quantitative representation of relative fold change in puromycin fluorescence intensity observed in Fig. 4C. Mean fluorescence intensity values were calculated from multiple fields acquired from tissue section for each sample. Data are presented as mean ± s.d, *p < 0.05. (**E**) Representative western blotting images depicting changes in mTOR phosphorylation in fasted and re-fed mice. (**F**) Representative images of WB-SUnSET analysis of heart tissues lysates from four, seven and ten months old mice. Ponceau staining was used to confirm equal loading. (**G**) Quantitative representation of puromycin incorporation observed in Fig. 4F. The results are expressed as the fold change relative to 4-month-old mice. n = 3–4 mice per group. Data are presented as mean ± s.d, *p < 0.05. (**H**) Representative images of WB-SUnSET analysis of mouse heart tissue from 2-month (young mice) and 24 months old (aged mice) mice. Ponceau staining was used to confirm equal loading. (**I**) Quantitative representation of puromycin incorporation observed in Fig. 4H. The results are expressed as the fold change relative to young mice. n = 6 mice per group. Data are presented as mean ± s.d, *p < 0.05.

We applied the principles of WB-SUnSET assay to track the changes in protein synthesis during the development of ISO induced hypertrophy. While the protein synthesis rates steadily increased till the third day of ISO administration, the rates declined on the fifth and the seventh day of ISO treatment (Fig. 4A,B). We observed a similar trend when we analyzed protein synthesis by IHC-SUnSET as well (Fig. 4C,D). The increased protein synthesis observed in the earlier stages of ISO treatment is critical for the establishment of hypertrophy and is mirrored by a concomitant increase in the cell size of cardiomyocytes (Fig. 3D and 3E). However, significant damage to cardiomyocytes due to chronic ISO treatment, evident from the H&E and Trichome staining might have contributed to the decrease in protein synthesis at the later stages (Fig. 3D,F and G). In addition, TUNEL assay revealed increased proportion of TUNEL positive cells on the fifth and the seventh day of ISO treatment indicating cardiomyocyte apoptosis (Supplementary Fig. 1A,B). This evidence further supports the observed reduction in the global protein synthesis in later stages of ISO treatment. Collectively, these results suggest that SUnSET assay can be useful to study changes in cardiac protein synthesis during pathological hypertrophy.

### SUnSET assay reliably measures the increase in rate of protein synthesis during postnatal cardiac growth and aging

The cardiomyocytes exit the cell cycle soon after birth and hence majority of the cardiomyocytes stop proliferating^29^. The primary mechanism by which the post-natal heart grows is through the enlargement of existing cardiomyocytes which is termed as postnatal or maturational hypertrophy^30^. Protein synthesis is one of the fundamental mechanisms that drives cellular growth. In our work, we intended to study the changes in the cardiac protein synthesis during the postnatal physiological growth of the mice. When the heart increases in size during organismal growth, the protein synthesis increases. In this context, we chose mice of three different age groups (four, seven and ten months old mice) spanning the first year after birth. Protein synthesis has been shown to vary rapidly in response to food intake^31–33^. The alterations in signaling pathways controlling protein synthesis following starvation and refeeding is well documented^31–35^. Our western results suggest that feeding activates mTOR signaling and might promote protein synthesis (Fig. 4E). The mice were starved for six hours and then fed for one hour to prevent any effects on protein synthesis due to differential feeding patterns. WB-SUnSET analysis of the heart tissues from four, seven, and ten-month-old mice revealed a steady increase in the protein synthesis rates (Fig. 4F,G). This suggests that increase in protein synthesis might facilitate the increase in the size of this muscular organ commensurate with the overall growth of the animal. However, it is well established that protein synthetic capacity decreases during aging of mice^36,37^. Therefore, we also measured the protein synthesis rates between young mice (2 months old) and aged mice (24 months old) by WB-SUnSET analysis. Our findings suggest that SUnSET assay can accurately measure the decreases in protein synthesis in aged animals (Fig. 4H,I), which was demonstrated by earlier works^36,37^. It further establishes the validity and adaptability of non-radioactive SUnSET assay to measure cardiac protein synthesis.

## Discussion

Sudden or sustained increase in hemodynamic load on the heart due to various physiological or pathological conditions culminates in a cardiac hypertrophic phenotype^38^. The progression of cardiac hypertrophy is marked by significant alterations in morphological parameters like size, shape and thickness of this muscular organ. Such changes are often brought about by changes in tightly regulated protein synthesis^39^. Thus, investigating changes in protein synthesis is critical in the context of hypertrophying heart. Our study provides strong evidence for the suitability of the non-radioactive SUnSET assay to measure changes in cardiac protein synthesis. This makes measuring changes in protein synthesis accompanying various cardiac remodeling processes simple, straightforward, risk-free and less expensive.

For decades, various radiolabeled tracers of amino acids such as ^3^H-leucine, ^3^H or ^14^C-phenylalanine, ^35^S-methionine and ^14^C-tyrosine have been extensively employed for monitoring protein synthesis^8,40^. Interestingly, the use of puromycin in measuring protein synthesis also has a long history. The incorporation of puromycin into elongating peptides finds mention as early as in the 1960s^10^. Since then multiple investigators have successfully employed ^3^H-labeled puromycin to study protein synthesis and turnover^41,42^. While high concentrations of puromycin is toxic or even lethal to cells, very low concentrations do not affect overall protein synthesis rates^9^. The recently introduced SUnSET method has greatly simplified the entire procedure of measuring protein synthesis. The entire procedure is nonradioactive and does not require any specialized medium such as in the case of incorporation of radiolabeled amino acids. After its description, multiple independent investigators have successfully adapted the SUnSET assay across diverse platforms. In addition, recent reports have also successfully applied the SUnSET assay to measure protein synthesis in the heart^5,43^.

We aimed to develop a simplistic and reliable method for measuring changes in cardiac protein synthesis associated with hypertrophying hearts. For this purpose, we used the β_1-_adrenergic receptor agonist ISO to demonstrate the usefulness of the SUnSET assay in monitoring *in vivo* cardiac protein synthesis. The catecholamine ISO is a well-established hypertrophic agent known to stimulate protein synthesis in heart^44^. We observed increased protein synthesis rate in hearts of ISO treated mice but not in skeletal muscle tissues of the same animal. These results agree with the previous reports and shows that the assay is highly specific for tissue specific changes^18^. Notably, the absence of any appreciable signal in samples from cycloheximide treated mice and the mice not injected with puromycin establishes the specificity of the anti-puromycin antibody. Further, similar results were observed when protein synthesis was analyzed by IHC-SUnSET, which shows the adaptability of the principle across multiple platforms. In addition, we also tracked protein synthesis under *in vitro* conditions using primary cultures of neonatal rat cardiomyocytes. We employed another hypertrophic agent, phenylephrine (α1-adrenergic receptor agonist) in addition to ISO for our *in vitro* studies. While both ISO and PE triggered significant increase in protein synthesis, Torin1, an ATP competitive inhibitor of mTOR effectively inhibited protein synthesis. These results agree with the previous reports and confirm the validity of this assay under the conditions employed^4,17,45^. Taken together these results clearly establish that both increase and decrease in protein synthesis can be accurately documented using this method in the cardiac milieu.

In summary, we report that the SUnSET assay can be used as a simple and a valid alternative to traditional methods of protein synthesis measurements in heart. Using the assay, we studied the changes in protein synthesis occurring during the development of agonist-induced cardiac hypertrophy. In addition, we also report that the cardiac protein synthesis increases at the earlier stages of life, but markedly decreased in aged mice.

## Materials and Methods

### Cardiomyocyte primary culture

Cardiomyocytes were isolated from 0–2 days old neonatal Sprague Dawley or Wistar rats and cultured on gelatin coated culture dishes according to our previously described protocol^46^. Briefly, the pups were anaesthetized with 1.0% isoflurane and sacrificed by decapitation. The hearts collected from the pups were digested enzymatically using a mixture of 0.2% trypsin, 0.4 mg/ml Collagenase Type II (both from Gibco) and 0.01 M of D-glucose prepared in PBS. Digestion was carried out for 5 min at 37 °C with gentle shaking at 250 rpm for multiple rounds. The unwanted erythrocytes and the debris were discarded from the first round of digestion. The single cell suspension from the subsequent rounds of digestion was collected in 100% horse serum (Gibco) and then plated on tissue culture polystyrene plates. After 1 hour of pre-plating, the non-adherent cell population enriched with cardiomyocytes were collected and seeded on gelatin coated culture dishes at appropriate cell density. The cardiomyocytes were maintained in high glucose DMEM (Sigma) supplemented with 10% fetal bovine serum (Gibco) and antibiotic-antimycotic mix (Gibco) and maintained in a humid 5% CO_2_ incubator at 37 °C. For measuring protein synthesis *in-vitro* by SUnSET assay, the cardiomyocytes were treated with 1 μM puromycin (P8833, Sigma) for 30 min prior to harvesting. For inhibition of global protein synthesis, the cells were treated with cycloheximide (Amresco 94271) at a final concentration of 100 µg/ml along with puromycin. For measuring relative changes in protein synthesis, healthy and contracting cardiomyocytes were treated with vehicle (150 mM NaCl and 1 mM acetic acid) or 20 μM isoproterenol (Sigma I6504, abbreviated as ISO) or 100 μM Phenylephrine (Sigma P6126, abbreviated as PE) for 24 hrs. Torin1 (Sigma) treatment was carried out at 250 nM for 6 hrs. Finally, the cells were pulsed with puromycin for 30 min and were harvested. The cells were lysed in ice cold 1 × cell lysis buffer (20 mM Tris-HCl pH7.5, 150 mM NaCl, 1 mM EDTA, 1 mM EGTA, 1% Triton, 2.5 mM sodium pyrophosphate, 1 mM sodium orthovanadate, 1 mM PMSF and 1 × protease inhibitor cocktail from Sigma), centrifuged at 12000 rpm for 10 min at 4 °C and the supernatant was collected.

### Animal studies

All animal studies were performed after due approval from the Institutional Animal Ethics Committee (IAEC), Indian Institute of Science, Bangalore, constituted as per the article number 13 of the Committee for the Purpose of Control and Supervision of Experiments on Animals (CPCSEA) rules, laid down by Government of India. All the studies conformed the Guide for the Care and Use of Laboratory Animals published by the US National Institute of Health (NIH Publication No. 85–23, revised 1996). Six weeks old, healthy and age matched swiss albino male mice were used for all the experiments. The animals were maintained on a regular chow diet in individually ventilated cages at the clean air facility. The mice were maintained under a standard 12-h light/dark cycle and had *ad libitum* access to food and water. For measuring protein synthesis by SUnSET assay, the mice were intraperitoneally injected with puromycin at a dose 40 nmol / g of body weight, and sacrificed after 30 min. For inhibition of global protein synthesis, the mice were injected with cycloheximide at a dose of 0.1 mg / g of body weight, 1 hour prior to administration of puromycin. For studying relative changes in protein synthesis, the mice were injected with either vehicle or two doses of isoproterenol at 5 mg/kg of body weight at 12-hour interval. The mice were then finally pulsed with puromycin, 24-hours post first dose of ISO and were sacrificed for analysis. For the development of experimentally induced hypertrophy, the mice were injected intraperitoneally either with vehicle or ISO, twice daily at a dose of 10 mg/kg of body weight. Cardiac function was assessed by measuring the ejection fraction by echocardiography using the Vevo® 1100 Imaging System (FUJIFILM VisualSonics) as described previously^24^. The animals were then sacrificed after pulsing them with puromycin for 30 min as described earlier and their organs were harvested, snap frozen in liquid nitrogen and stored in −80 °C until further use. Heart weight and tibia length were measured using a standard weighing balance and a Vernier caliper respectively. Tissue lysates were prepared from frozen tissues using the Polytron homogenizer (Kinematica) in ice-cold tissue lysis buffer (40 mM Tris, pH 7.5; 1 mM EDTA; 5 mM EGTA; 0.5% Triton X-100; 25 mM β-glycerophosphate; 25 mM NaF; 1 mM Na_3_VO_4_; 10 μg/ml leupeptin; and 1 mM PMSF), and the cleared homogenate was used for further analysis.

### Western blot SUnSET (WB-SUnSET)

The cardiomyocyte lysates and the tissue homogenates were normalized for equal amounts of protein using the Bradford method. The samples were then boiled with Laemmli Sample Buffer (Bio-Rad) supplemented with 5% β-mercaptoethanol at 96^0^C for 5 min and electrophoresed on a 10% SDS-Polyacrylamide gel at constant voltage. The proteins were then transferred onto a 0.45 µm PVDF membrane (Amersham Hybond, GE). Blocking was carried out for 1 hr with 5% milk prepared in Tris-Buffered Saline with 0.05% Tween 20 (TBST). The blots were then washed thrice in TBST and incubated with primary antibody prepared in 5% BSA in a rocker at 4°C overnight. The membranes were then washed thrice with TBST and incubated with HRP conjugated secondary antibody (Clean-Blot IP Detection Reagent, Thermo Scientific) prepared in 1% milk. Chemiluminescent signals were detected using BioRad Clarity ECL Western Blotting Substrate and the images were acquired using a chemiluminescence imager.

### Immunohistochemistry SUnSET (IHC-SUnSET) and Immunofluorescence SUnSET (IFC-SUnSET)

For immunohistochemical and histopathological evaluation, cardiac tissues harvested from the mice were fixed in 10% neutral buffered formalin for 72 hrs at room temperature. The procedures carried out here were adapted from our previous work^23^. Briefly, for the measurement of cell size, the heart sections were stained with 10 μM wheat germ agglutinin (WGA) and the images were obtained using confocal microscopy. The cell size of myocytes was measured using the NIH ImageJ software. Fibrosis was detected using the standard Masson’s trichrome staining. The sections were scored for fibrosis on a scale of 1 to 5 with 5 indicating maximum fibrosis. Hematoxylin and eosin (H&E) staining was carried out using the standard methods and the tissue sections were examined for histopathological changes under the light microscope. Scoring for immune cell infiltration was done on a scale of 1 to 5 based on H&E staining with 5 indicating the maximum infiltration. TUNEL assay was performed on paraffin embedded cardiac tissue sections according to the manufacturer’s instructions using the *in situ* apoptosis detection kit (Abcam). For immunohistochemical (IHC) examination, 5 µm thick formalin-fixed paraffin-embedded tissue sections of heart were mounted on poly-L-lysine coated slides, air dried for 30 min, and incubated with primary antibody (PMY-2A4, DSHB) diluted to 1:100 in 1% BSA prepared in Phosphate-Buffered Saline (PBS) for 2 hours at room temperature. Secondary antibody was used at a dilution of 1:200 diluted in PBS and incubated for 1 h. The nuclei were stained using Hoechst 33342 (1:2000 dilution in PBS). For immunofluorescence experiments (IFC-SUnSET), cardiomyocytes were grown on gelatin coated coverslips, fixed with 4% formalin for 15 min and permeabilized with 0.2% Triton X-100 for 5 min. The primary and secondary antibodies were prepared in 1% BSA prepared in Phosphate-Buffered Saline with 0.02% Tween 20 (PBST). For ANP staining ANP from Abcam (ab14348) was used at a dilution of 1:300. Secondary antibodies were used at a dilution of 1:400. Images were acquired using the LSM 770 or LSM 880 confocal microscope.

### Real-time qPCR analysis

Real time qPCR was performed as described previously^46^. The following primer sequences were used: Atrogin-1 For: AGCGCTTCTTGGATGAGAAA, Atrogin-1 Rev: GGCAGTCGAGAAGTCCAGTC; MuRF-1 For: TGCCTGGAGATGTTTACCAAGC, MuRF-1 Rev: AAACGACCTCCAGACATGGACA; Actin For: TTCTACAATGAGCTGCGTGTG, Actin Rev: GGGGTGTTGAAGGTCTCAAA.

### [^3^H]-Leucine incorporation assay

The procedure was adapted and modified from our previously described protocol^19^. Briefly, the cardiomyocytes treated with either vehicle or ISO were incubated with [^3^H]-leucine (1.0 mCi/ml). Cells were then washed with phosphate-buffered saline and 10% trichloroacetic acid was added to precipitate the proteins. The resultant pellet was solubilized in 0.2 N NaOH and diluted with one-sixth volume of scintillation fluid. The radioactivity was measured in a scintillation counter and the values were normalized with DNA content and measured using the Qubit dsDNA HS assay kit.

### Statistical analysis

Statistical analyses were performed using the GraphPad Prism software. Pairwise comparisons were performed using t-tests and one-way ANOVA. ZEN and ImageJ software were used for analyzing confocal images. Quantification of western blots was performed using the ImageJ software.

### Data availability

The data generated during and/or analyzed during the current study are available from the corresponding author on reasonable request.

## Electronic supplementary material

Supplement file 1
