# Supplementary material for: Systematic evaluation of the adaptability of the non-radioactive SUnSET assay to measure cardiac protein synthesis
Source: Sci Rep. 2018 Mar 15;8:4587. doi: 10.1038/s41598-018-22903-8 (PMC5854694; doi:10.1038/s41598-018-22903-8)
Supplement: Supplementary file 1 — Supplement file 1 [file 41598_2018_22903_MOESM1_ESM.pdf]

Systematic evaluation of the adaptability of the non-radioactive SUnSET assay to measure cardiac protein synthesis

Venkatraman Ravi[1], Aditi Jain[2], Faiz Ahamed[1], Nowrin Fathma[1], Perumal Arumugam Desingu[1], Nagalingam R. Sundaresan[1,2 #]

[1] Cardiovascular and Muscle Research Laboratory, Department of Microbiology and Cell Biology, Indian Institute of Science, Bengaluru, India

[2] Centre for BioSystems Science and Engineering, Indian Institute of Science, Bengaluru, India

Supplementary Figure 1:

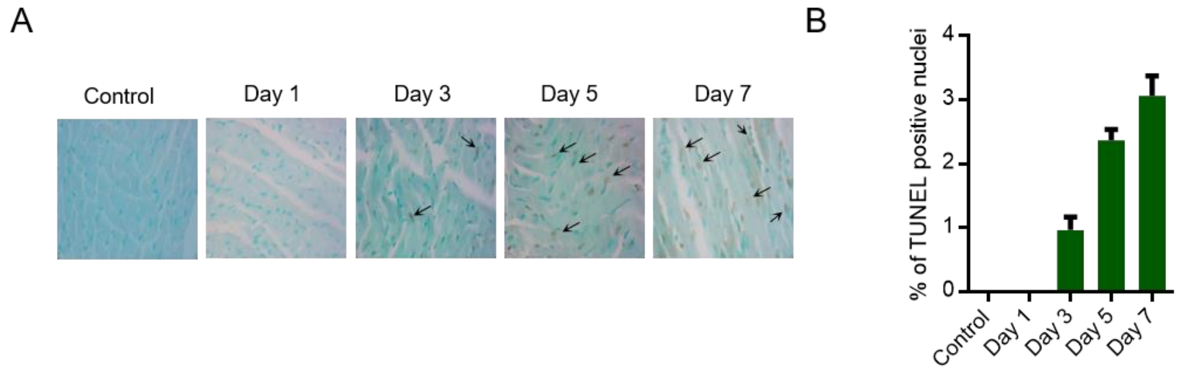

A) Representative images of TUNEL assay performed in heart tissues sections of ISO injected mice for different time periods between 0-7 days. The black arrows indicate TUNEL positive apoptotic nuclei. B) Quantification of TUNEL positive nuclei expressed in percentage. n=3 animals, 1000 nuclei were counted for each sample.
